# Invasive lobular carcinoma of the breast detected with real-time virtual sonography: a case report

**DOI:** 10.1186/s40792-023-01667-y

**Published:** 2023-05-19

**Authors:** Yukie Ito, Kimihito Fujii, Masayuki Saito, Hirona Banno, Mirai Ido, Manami Goto, Takahito Ando, Yukako Mouri, Junko Kousaka, Tsuneo Imai, Shogo Nakano

**Affiliations:** Division of Breast and Endocrine Surgery, Department of Surgery, Aichi Medical University, 1-1, Yazako-Karimata, Nagakute-City, Aichi 480-1195 Japan

**Keywords:** MRI, Non-mass-enhancement lesion, RVS, Invasive lobular carcinoma

## Abstract

**Background:**

Invasive lobular carcinoma (ILC) sometimes presents with unique clinical, pathologic, and radiographic features. In this case report, we describe a patient with ILC, whose initial presentation consisted with symptoms secondary to bone-marrow dissemination. In addition, the breast primary was revealed only by magnetic resonance imaging (MRI) followed by real-time virtual sonography (RVS).

**Case presentation:**

A 51-year-old woman presented to our outpatient clinic with dyspnea on exertion. She had severe anemia (hemoglobin, 5.3 g/dL) and thrombocytopenia (platelet count, 31 × 10^3^/mL). Bone-marrow biopsy was performed to evaluate hematopoietic function. The pathologic diagnosis was bone-marrow carcinomatosis due to metastatic breast cancer. Initial mammography followed by ultrasonography (US) failed to detect the primary tumor. On MRI, a non-mass-enhancement lesion was observed. While second-look US also did not detect the lesion, it was clearly visualized with RVS. We were finally able to biopsy the breast lesion. The pathologic diagnosis was ILC positive for both estrogen receptor and progesterone receptor, with 1 + immunohistochemical staining for human epidermal growth factor receptor 2. This case of ILC was characterized by bone-marrow metastasis. Due to decreased cell adhesion, the risk of bone-marrow metastasis is higher in ILC than in invasive ductal carcinoma, the most prevalent type of breast cancer. Biopsy of the primary lesion, which was initially only detected with MRI, was successfully performed with clear visualization during RVS, which is based on the fusion of MRI and US images.

**Conclusion:**

In this case report and literature review, we describe the unique clinical characteristics of ILC and a strategy for identifying primary lesions that are initially only visualized with MRI.

## Background

Invasive lobular carcinoma (ILC) is the second most common type of invasive breast cancer, after invasive ductal carcinoma (IDC). ILC sometimes presents with unique clinical, pathologic, and radiographic features [1, 2]. In this case report, we describe a patient with ILC in whom the primary lesion was difficult to detect. Initial presentation consisted with symptoms secondary to bone-marrow dissemination. Real-time virtual sonography (RVS) was ultimately used to identify the primary breast lesion. RVS played an important role in making the precise pathologic diagnosis [3].

## Case report

**Fig. 1:**
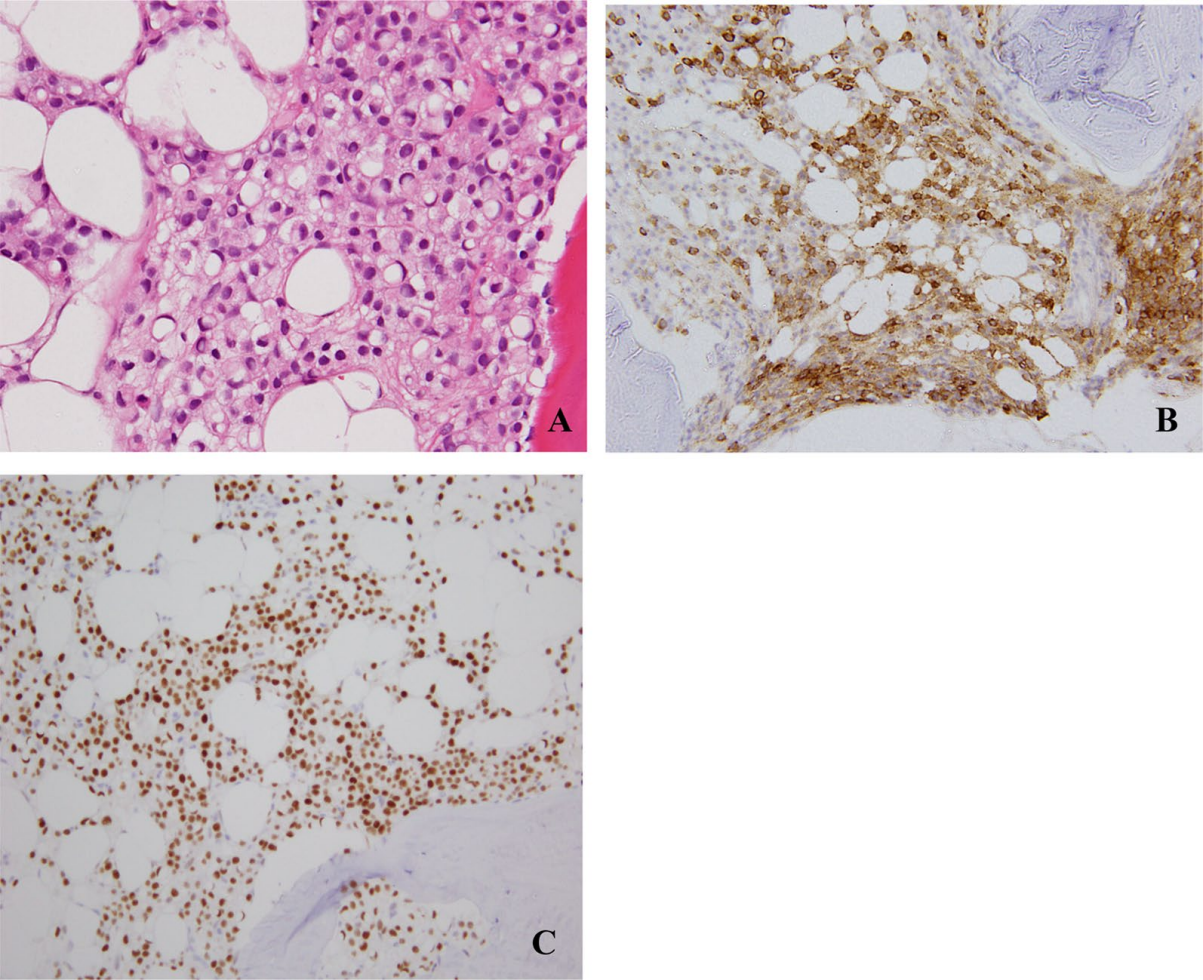
Pathological findings in the bone-marrow biopsy specimen. **A** Hematoxylin–eosin staining. Signet-ring cell carcinoma cells were widespread. There were very few hematopoietic cells. **B** Positive immunohistochemical staining with an anti-mammaglobin A antibody, indicating that breast cancer was the primary tumor. **C** Positive immunohistochemical staining with an anti-estrogen receptor antibody. (Magnification × 20 for all.)

**Fig. 2:**
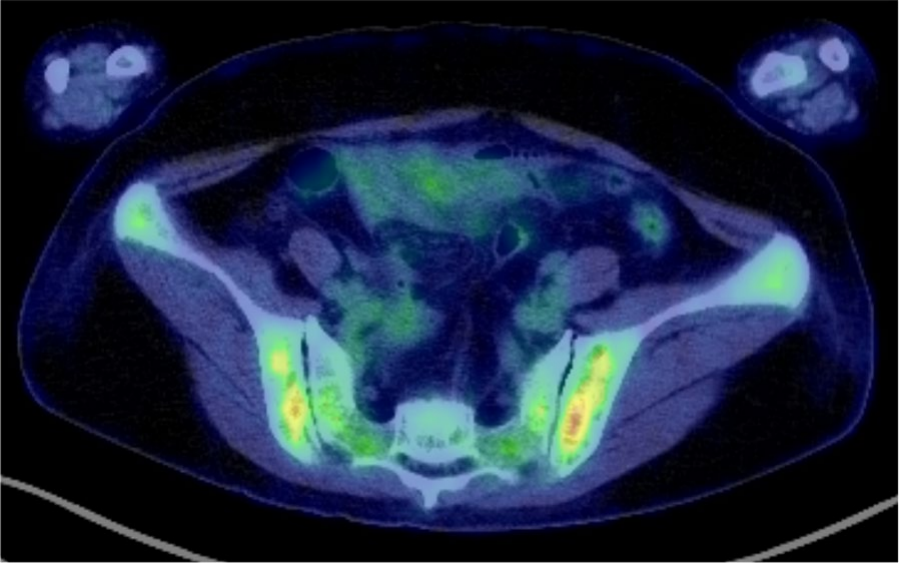
In the PET–CT scan image around the pelvis, diffuse FDG uptake is demonstrated

**Fig. 3:**
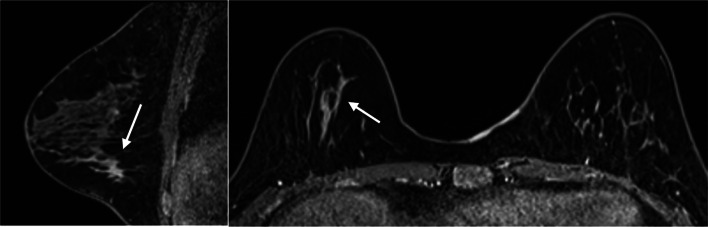
Contrast-enhanced prone MRI depicted a region with NME in the right breast (arrow)

**Fig. 4:**
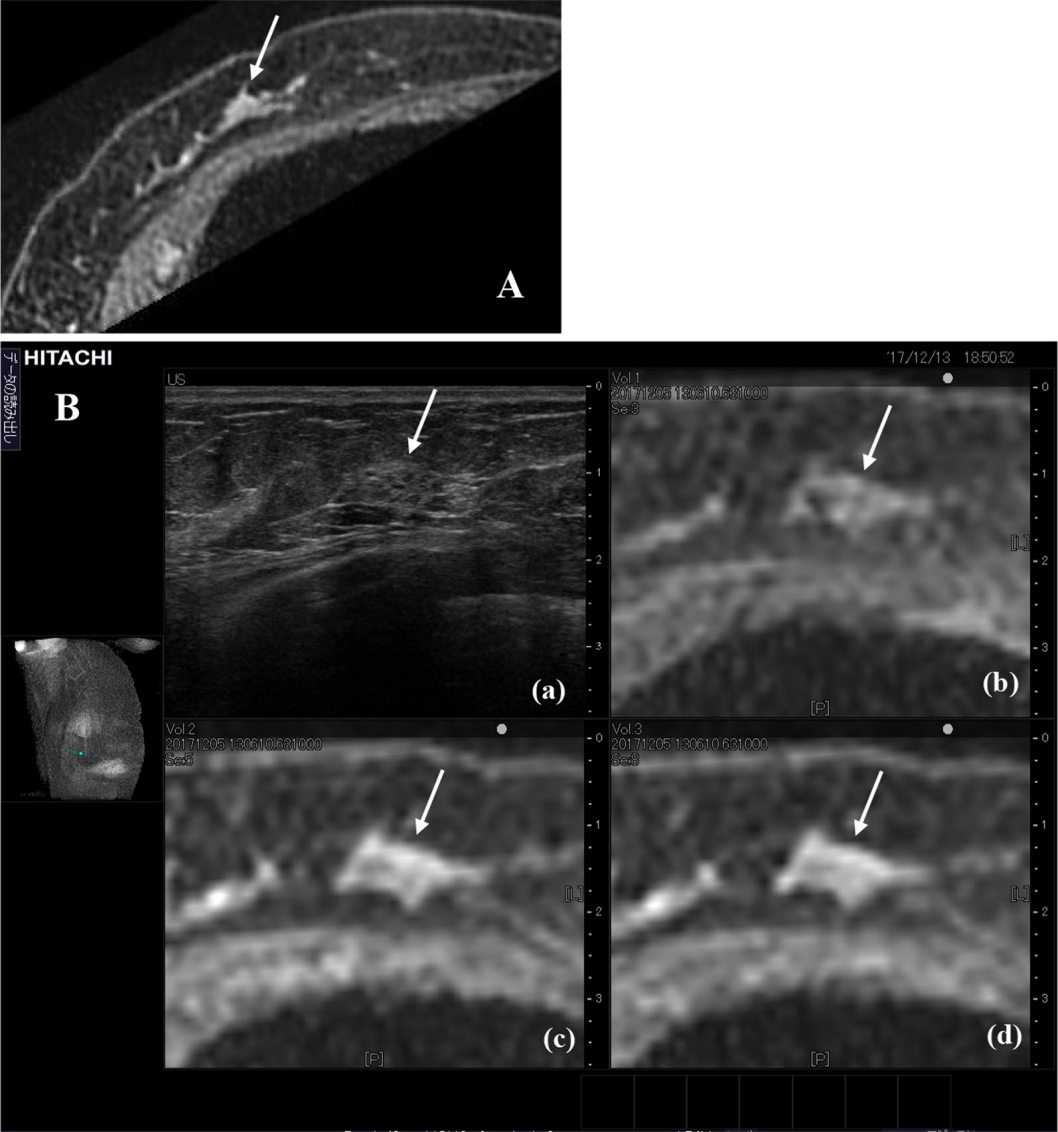
**A** T1-weighted, contrast-enhanced additional supine MRI with multi planar reconstruction (MRI-MPR) showed an irregular enhancing mass (arrow). **B** View from the RVS monitor. Sonography showed a heterogeneous mass with low echogenicity and irregular shape. A pre-contrast MRI-MPR image is shown in (b). An early phase MRI-MPR image is shown in c. A late-phase MRI-MPR image is shown in d. Images (b–d) correspond to sonographic cutaway images in a

A 51-year-old woman presented with dyspnea on exertion that had started 2 weeks earlier. Physical examination revealed only conjunctival pallor. Breast palpation revealed no obvious masses. Laboratory examination demonstrated severe anemia, with a hemoglobin level of 5.3 g/dL. Since the patient also had a very low platelet count of 31 × 10^3^/mL, bone-marrow biopsy was performed to evaluate hematopoietic function. Pathological examination revealed bone-marrow carcinomatosis with widespread metastatic signet-ring cell carcinoma cells and very few hematopoietic cells. Immunohistochemical staining yielded positive results for mammaglobin A (Cosmo Bio, Tokyo, Japan) and estrogen receptor (Roche Diagnostics, Basel, Switzerland), indicating that the metastases had originated in the breast (Fig. 1). Neither mammography nor ultrasonography (US) revealed the primary tumor. Positron emission tomography–computed tomography (PET–CT) demonstrated bone-marrow metastases with diffuse ^18^F-Fluorodeoxyglucose (FDG) accumulation in both vertebral bodies and pelvis (Fig. 2). Magnetic resonance imaging (MRI) of both breasts finally showed a non-mass-enhancement (NME) lesion on the right side that was suspected to be the primary tumor (Fig. 3). Neither second-look US nor US with elastography could reveal the lesion detected with MRI. MRI was one and only modality that could depict the primary breast cancer lesion. We, therefore, applied the RVS technique, which clearly revealed the primary cancer as a diffuse area of low echogenicity (Fig. 4). Core needle biopsy was finally performed. The pathologic diagnosis was ILC that was positive for both estrogen receptor and progesterone receptor, with 1 + immunohistochemical staining for human epidermal growth factor receptor 2 and a Ki-67 index of 5%.

We prefer chemotherapy as a primary therapy, because the cancer had spread systemically. Chemotherapy was started, along with red blood cell and platelet transfusions. The chemotherapeutic regimen consisted of intravenous paclitaxel 60 mg/m^2^ on days 1, 8, and 15 and intravenous bevacizumab 10 mg/kg on days 1 and 15, every 4 weeks. After 8 months of aggressive chemotherapy, the hemoglobin level and platelet count returned to normal. Levels of the tumor marker carbohydrate antigen 15–3, decreased from 1105 to 780 U/mL (normal range, < 35 U/mL). Anastrozole was initiated as adjuvant hormonal treatment. Approximately a year has passed since the initial cancer diagnosis with no progression identified.

## Discussion

ILC is the most common of the special types of breast cancer. ILC accounts for up to 15% of all breast cancer. It has a unique metastatic presentation, with a predilection for common sites such as the liver, lung, and bone as well as the gastrointestinal tract and gynecologic organs. ILC is also characterized by the lack of E-cadherin, a tumor suppressor that plays an important role in epithelial cell–cell adhesion and tumor morphogenesis. Loss of E-cadherin expression leads to decreased cellular adhesion, resulting in cell migration and metastatic spread [2, 4, 5]. These distinctive features of ILC presumably led to the bone-marrow metastasis described in the current report. Borst et al. reported that the metastatic pattern of ILC differs from that of IDC. The rate of bone-marrow metastasis was 21.2% among 359 patients with ILC and 14.4% among 2,246 patients with IDC, a statistically significant difference [6]. The prognosis of bone-marrow metastasis is so poor that chemotherapy should be considered. We began treatment of our patient with paclitaxel plus bevacizumab. Her disease status improved sufficiently. Of note, initial therapy for metastatic breast cancer with paclitaxel plus bevacizumab was previously shown to prolong progression-free survival [7].

In the present case, the primary ILC lesion in the breast was only revealed with MRI based on characteristic NME. As ILC invades the adjacent breast tissue along mammary ducts and usually does not form a palpable lump, conventional mammographic and ultrasound challenges result in false-negative diagnoses [8]. Brem et al. reported that the sensitivity of mammography, US and MRI in the detection of ILC among 28 biopsy-proven cases were 79%, 68% and 83%, respectively, indicating that the MRI presented the highest sensitivity [9]. In the fifth edition of the American College of Radiology Breast Imaging Reporting and Data System (BI-RADS) lexicon, NME is classified as homogeneous, heterogeneous, clumped, and in a clustered ring [10]. NME lesions are considered to indicate malignancy; however, the prevalence of NME is much lower than that of mass enhancement [11, 12]. The BI-RADS lexicon has been shown to be inadequate for distinguishing between benign and malignant NME lesions [13–15]. In an analysis of 99 benign and 30 malignant NME lesions, Aydin reported that 17 (13.2%) lesions had a segmental distribution, like our patient’s lesion, of which 5 were benign and 12 were malignant [11]. Segmentally distributed lesions are most likely to be malignant.

In our case, second-look US failed to visualize the primary lesion in the breast that was detected by MRI. RVS was adopted as the next modality, which successfully demonstrated the lesion as a heterogeneous mass with low echogenicity and an irregular edge. It was previously reported that second-look US of breast cancer lesions detected with MRI identified 49% of mass lesions, 42% of focal lesions, and 15% of NME lesions [16]. RVS can be effective for clear visualization of NME lesions. The RVS system employed in this case simultaneously displayed both sonographic and MRI cutaway images of the same site in real time, which has excellent accuracy for identifying breast lesions with enhancement on MRI [3]. Nakano et al. also reported that RVS was highly useful for demonstrating NME lesions detected with MRI in 12 patients with breast cancer who underwent breast-conserving surgery [17].

For pathological confirmation of the lesions visualized only by MRI, MRI-guided biopsy has become increasingly available. However, these techniques are not commonly available and require the costly use of the device and the medical staffs. On the other hand, RVS does not require large equipment and can be carried out easily at any time, suggesting that RVS is a patient-friendly technique that can visualize a large proportion of MRI findings. Furthermore, it should be validated that RVS could select the patients that really undergo MRI-guided biopsy. Nakano et al. reported that the detection rates of MRI-detected lesions with second-look sonography using RVS or not were 90% and 30%, respectively (*P* < 0.001) [18]. There would be thoughts that the MRI-guided biopsy is applied for the lesions that RVS failed to visualize. Unfortunately, no direct comparison between MRI-guided and RVS-guided biopsy has been demonstrated. The comparison of vacuum-assisted biopsy (VAB) among MRI, stereotactically and ultrasound-guided was reported by Imschweiler et al. [19]*.* In that report, the technical success rates of MRI-guided, stereotactically guided and ultrasound-guided VAB were 98.4%, 99.1% and 99.6%, respectively. There was a significant difference in the technical success rates between the MRI and ultrasound-guided VAB (*P* < 0.001). The complications were hemorrhage, infection, lesion miss and so forth. The total complication rate of ultrasound-guided VAB was significantly lower than that of MRI-guided VAB (*P* < 0.001).

RVS technique have some limitations. First, the MRI data introduced into RVS system are constructed with supine-position MRI examination, which is not established method for diagnosing breast cancer. Second, the US examinations are considered to be poor at reproducibility, because the breast will change its shape easily and the quality of the examination depend on the examiner’s skill. The result of multi-institutional study which was conducted for further investigation of the role of RVS and the examination of the interinstitutional reproducibility is awaited.

## Conclusion

We encountered a patient with ILC and bone-marrow metastasis in which the initial chemotherapeutic intervention was effective. The primary breast cancer, which was identified as an NME lesion with MRI, could be clearly visualized with RVS and those radiological findings were considered to be relevant to morphological feature of ILC. Biopsy of the primary lesion was successfully performed by RVS-guidance. For the treatment of breast cancer, detailed imaging techniques and precise pathologic diagnosis are both essential.

## Data Availability

The data that support the findings of this study are available on request from the corresponding author. The data are not publicly available due to ethical restrictions.

## Abbreviations

ILC: Invasive lobular carcinoma
IDC: Invasive ductal carcinoma
RVS: Real-time virtual sonography
US: Ultrasonography
MRI: Magnetic resonance imaging
NME: Non-mass-enhancement
FDG: ^18^F-Fluorodeoxyglucose
PET-CT: Positron emission tomography–computed tomography
MPR: Multi planar reconstruction
BI-RADS: Breast Imaging Reporting and Data System
